# Investigating Patients’ Intention to Continue Using Teleconsultation to Anticipate Postcrisis Momentum: Survey Study

**DOI:** 10.2196/22081

**Published:** 2020-11-26

**Authors:** Antoine Grenier Ouimet, Gerit Wagner, Louis Raymond, Guy Pare

**Affiliations:** 1 Research Chair in Digital Health, HEC Montreal Montreal, QC Canada; 2 Université du Québec à Trois-Rivières Trois-Rivières, QC Canada

**Keywords:** teleconsultation, online medical consultation, remote consultation, continuance intention, COVID-19, telemedicine, survey research

## Abstract

**Background:**

The COVID-19 crisis has drastically changed care delivery with teleconsultation platforms experiencing substantial spikes in demand, helping patients and care providers avoid infections and maintain health care services. Beyond the current pandemic, teleconsultation is considered a significant opportunity to address persistent health system challenges, including accessibility, continuity, and cost of care, while ensuring quality.

**Objective:**

This study aims at identifying the determinants of patients’ intention to continue using a teleconsultation platform. It extends prior research on information technology use continuance intention and teleconsultation services.

**Methods:**

Data was collected in November 2018 and May 2019 with Canadian patients who had access to a teleconsultation platform. Measures included patients’ intention to continue their use; teleconsultation usefulness; teleconsultation quality; patients’ trust toward the digital platform, its provider. and health care professionals; and confirmation of patients’ expectations toward teleconsultation. We used structural equation modeling employing the partial least squares component-based technique to test our research model and hypotheses.

**Results:**

We analyzed a sample of 178 participants who had used teleconsultation services. Our findings revealed that confirmation of expectations had the greatest influence on continuance intention (total effects=0.722; *P*<.001), followed by usefulness (total effects=0.587; *P*<.001) and quality (total effects=0.511; *P*<.001). Usefulness (β=.60; *P*<.001) and quality (β=.34; *P*=.01) had direct effects on the dependent variable. The confirmation of expectations had direct effects both on usefulness (β=.56; *P*<.001) and quality (β=.75; *P*<.001) in addition to having an indirect effect on usefulness (indirect effects=0.282; *P*<.001). Last, quality directly influenced usefulness (β=.34; *P*=.002) and trust (β=.88; *P*<.001). Trust does not play a role in the context under study.

**Conclusions:**

Teleconsultation is central to care going forward, and it represents a significant lever for an improved, digital delivery of health care in the future. We believe that our findings will help drive long-term teleconsultation adoption and use, including in the aftermath of the current COVID-19 crisis, so that general care improvement and greater preparedness for exceptional situations can be achieved.

## Introduction

### Background

The COVID-19 pandemic has heightened the urgency of delivering health care through digital technologies [1,2], contributing momentum to existing efforts of transitioning to models of digitally mediated health care provision. As people are encouraged to avoid public places and institutions, telemedicine appears particularly attractive for limiting the propagation of the virus while ensuring the continued care of patients deemed at risk [1,3-5]. One especially promising telemedicine technology is teleconsultation, which has been considered “capable of reducing emergency room visits, conserving health care resources, and avoiding the spread of COVID-19 by treating patients remotely” [2].

Although the current crisis has induced a high demand, we focus on postpandemic use of teleconsultation and recognize its value for normal conditions. Teleconsultation, or a virtual visit, allows patients to consult with their health care providers online through devices that support text chat and videoconferencing [6]. This type of care delivery is often considered an opportunity to increase accessibility, continuity, and efficiency of care; achieve higher patient satisfaction; and reduce costs [6-9]. At the same time, teleconsultation is potentially comparable, or even preferable, to in-person visits within appropriate medical contexts [9,10].

Prior to the current public health crisis, teleconsultation’s general rate of adoption remained low [11-13]. The current situation has caused a profound spike in demand for digitally mediated consultation, and policy makers are breaking down several barriers to foster widespread adoption [14]. As a result, demand for teleconsultation has soared around the world. For instance, the number of teleconsultations in France increased approximately 50-fold in the weeks after it was hit by the pandemic [15].

With the increased demand for teleconsultation observed over the past months, it is an open question whether this momentum will lead to continued adoption in the long term, or whether patients are likely to abandon teleconsultation platforms once the crisis has passed. Previous research has investigated the initial adoption and use of teleconsultation (eg, [12,13,16,17]) but fails to provide explanations of patients’ continuance behavior of teleconsultation (ie, the “long-term or sustained use of an IT [information technology] by individual users over a period of time” [18]). Our lack of understanding of the postadoption behaviors of teleconsultation users is troubling since consumers often abandon the use of digital health apps and smart devices shortly after they first use them [19-21]. Given that materialization of the benefits of a technology relies on its effective and continuous use [22], this dearth of literature is significant in the context of teleconsultation platforms.

Considering these factors, this study aims to answer the following research question: What factors influence a patient’s intention to continue using a teleconsultation platform? To address this question, we draw on theories of information systems (IS) adoption and factors specific to teleconsultation services. We empirically tested our research model by surveying employees of two major Canadian companies who have access to the Dialogue teleconsultation platform.

### Research Model and Hypotheses Development

**Figure 1:**
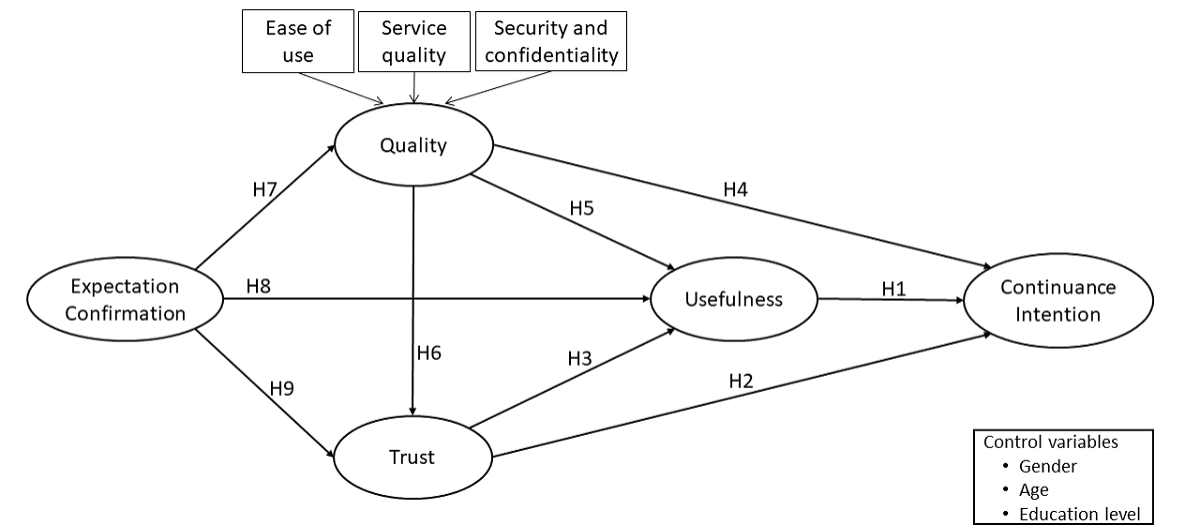
Research model. H: hypothesis.

To develop our research model (see Figure 1), we draw inspiration from IS research on continuance intention (CI) [23]. We searched the CI literature of the last three years (2015-2017) for peer-reviewed papers [23,24] through three databases: ABI/INFORM, Science Direct, and Web of Science. The search terms were: (“information systems” OR “IS” OR “information technology” OR “IT”) AND (“continuance intention”). We identified 221 papers matching our criteria. Specifically, our search of the literature and the descriptive literature review by Nabavi et al [23] resulted in five constructs relevant to the study of teleconsultation continuance by patients: CI, usefulness, quality, trust, and expectations confirmation. In a nutshell, our model contends that users’ initial expectations toward an IT are either confirmed or disconfirmed through the user experience [22]. This assessment is captured through the confirmation expectations construct. If the perceived performance of the teleconsultation platform exceeds users’ initial expectations, confirmation is said to be positive. In contrast, expectations that are higher than perceived performance produce a negative confirmation. This assessment influences users’ expectations toward their future experiences (ie, usefulness, quality, and trust), further affecting their intention to continue using the system [22]. As we aim to explore patients’ motivations to continue using teleconsultation, this approach enabled us to focus on the postadoption stage while capturing the preadoption effects of patients’ expectations within the use assessment phase of our model.

Using CI as a proxy to the actual continuous use behavior is a common practice in IT postadoption research [23]. This is in accordance with research on reasoned and plan behaviors in social psychology [18]. Hence, using CI as our dependent variable is consistent with past work and with our goal of identifying continuous teleconsultation use determinants. In this regard, the perceived usefulness of an IT, described as users’ perceptions of the benefits associated with its use [22], has been considered as one of the principal attitudinal beliefs explaining CI [23]. This relationship has been confirmed extensively in the extant literature and in health IT contexts related to teleconsultation such as mobile health (mHealth) [19,25,26]. Considering this, our first hypothesis (H) is as follows:

- H1: Patients’ intention to continue using teleconsultation is positively influenced by its usefulness.

IT users’ behavioral intentions have also been shown to depend on users’ level of trust with a technology and its provider [26-29]. Trust is defined as a party’s willingness to be vulnerable to the actions of a second party, whereas the latter is expected to act in the interests of the former, regardless of the former’s ability to monitor or control the latter [30]. In other words, trust is the *expectation* that a teleconsultation provider (ie, the Dialogue company), the health professional who assists the patient during the teleconsultation session, and the teleconsultation application can all be counted on. This factor is particularly critical in a virtual environment with high privacy and security risks [28]. These risks are present when users of medical teleconsultation systems disclose information about their physical or mental health, especially since they depend on and have no control over the health care provider [31]. As demonstrated in previous empirical studies, the usefulness of a technology is shaped by other expectations toward it, such as ease of use [32,33]. Therefore, we postulate that trust in a teleconsultation system and its provider affects its usefulness (ie, a teleconsultation service that is deemed untrustworthy is bound to be perceived as having little utility). Hence, we propose the following hypotheses:

- H2: Patients’ intention to continue using teleconsultation is positively influenced by their trust in it.
- H3: Usefulness of teleconsultation is positively influenced by patients’ trust in it.

Previous empirical evidence indicates that the overall quality of a health care service is of primary importance in shaping the behavioral intent of its users [26,34,35]. Teleconsultation quality is defined here as a patient’s judgment of the overall excellence of a teleconsultation. If virtual care is perceived as being of poor quality, it may discourage patients from using it and lead them toward other alternatives such as in-person health services [26]. Much like the assumption that a system that is difficult to use is perceived to be of little usefulness [32], and along with our rationale endorsing the positive influence of trust on usefulness, we postulate usefulness is also affected by the quality of the teleconsultation. In other words, a teleconsultation service perceived as being of poor quality is likely to be regarded as having little utility. In addition, empirical findings demonstrate that users’ trust is influenced by the perceived overall quality of the IT service (eg, [26,36]). Hence, we formulate the following hypotheses:

- H4: Patients’ intention to continue using teleconsultation is positively influenced by its overall quality.
- H5: Usefulness of teleconsultation is positively influenced by its overall quality.
- H6: Patients’ trust in teleconsultation is positively influenced by its overall quality.

Dagger et al [34] found that “customers evaluate service quality at an overall level, a dimensional level, and at sub-dimensional level and that each level drives perceptions at the level above.” As a result, the operationalization of this construct should be multidimensional and its measurement formative. Quality has also been theorized as a multidimensional construct in IS research (eg, [37,38]), and we, therefore, look to this field for inspiration to define service quality, system security and confidentiality, and ease of use as first-level dimensions of a teleconsultation quality. Service quality reflects the importance of the service provided to patients [38]. Confidentiality is the degree to which patients’ personal information will not be shared or used against their wishes [39]. Security is defined as patients’ perception of another party’s ability to protect their private information against loss and unwarranted access [40]. Prior studies have integrated security with constructs like privacy (eg, [41]), and thus, we combined security and confidentiality in a single construct. Ease of use, the degree to which an individual believes that an IT system can be used effortlessly [32], was also incorporated as a formative dimension of quality. Hence, we argue that an increase in the aforementioned dimensions would elevate the perception of the teleconsultation quality for its users and not the opposite. Accordingly, we defined a teleconsultation quality as a second-order construct composed of service quality, system security and confidentiality, and ease of use.

Following findings from the expectation confirmation model (ECM), we included the confirmation of expectations construct in our model. This construct refers to the perceived congruence between the user’s initial expectations of the IT use and its actual performance [22]. Its inclusion aims at enabling the comparison of users’ initial expectations regarding teleconsultation against the perceived performance resulting from its use. Since usefulness, quality, and trust are expectations toward medical teleconsultation, we posit the following hypotheses:

- H7: Quality of teleconsultation is positively influenced by the confirmation of patients’ initial expectations.
- H8: Usefulness of teleconsultation is positively influenced by the confirmation of patients’ initial expectations.
- H9: Patients’ trust in teleconsultation is positively influenced by the confirmation of their initial expectations.

Given the possibility that various demographic characteristics may influence the attitudes and use behaviors of health IT users (eg, [20,26]), the patients’ gender, age, and education level were included as control variables in the research model. Note that socioeconomic variables were not included in the research model given that all employees in the surveyed companies had free access to Dialogue’s teleconsultation services as part of their employment insurance benefits. Our research model is presented in Figure 1, and the definitions of the variables included in the model are provided in Multimedia Appendix 1.

## Methods

### Empirical Setting

The technology studied is the Dialogue teleconsultation platform, which was launched in spring 2016. This teleconsultation service, which is distributed to organizational clients, provides remote health care services to their employees and their employees’ relatives (ie, spouse and children). The platform allows patients to communicate with various health care professionals through chat or video calls. Health care professionals can refill prescriptions remotely, refer patients to an external specialist, assist them in navigating the public health care system, and provide medical follow-up from the Dialogue team. During a session, the patient first interacts with an artificial intelligence–based system that carries out an initial assessment of their needs and redirects the query accordingly. If the system does not refer the patient to another health service, a registered nurse continues the process and discusses the patient’s state of health in more depth. This step has three outcomes: immediate diagnosis of the patient, transfer of the patient to a Dialogue physician or nurse for consultation (via chat or video call), or redirection of the patient to a physical examination provided by another health care provider. Ultimately, the patient can either be diagnosed by a Dialogue nurse or physician, or recommended to another health provider that is more suitable to their needs.

The research model was empirically tested with a sample of Dialogue users at two large Canadian companies. The first one is a financial institution with approximately 2000 employees, most of whom are in Quebec. The second company specializes in marketing and advertising, and has close to 500 employees located in different provinces across Canada. At the time of the survey, the 2370 employees (1875 for the first company and 495 for the second) in these two companies had free access to Dialogue teleconsultation services. Both organizations are known for their innovative workplace health management practices, and their staff had access to the teleconsultation platform for over a year before data collection started.

### Data Collection

Given our main objectives, we conducted a cross-sectional survey study. The questionnaire was developed and administered using Qualtrics online survey software. The survey was administered in both French and English. Following the design of the questionnaire instrument, a pretest was conducted with 6 users of the teleconsultation system (4 French-speaking and 2 English-speaking users). As a result of the pretest, a few minor changes were made to the questionnaire, and an open-ended question about expectations confirmation was added. All study procedures were approved by the HEC Montreal’s research ethics committee.

### Operationalization of the Research Variables

The operationalization of the research variables was based on the extant literature. Given the maturity of the research stream, this approach benefits from previous empirical validations and, thus, ensures the validity and reliability of the selected measurement items. Indeed, all the selected items demonstrated appropriate psychometric qualities in prior studies. All items were based on a 5-point Likert scale, ranging from “totally disagree” to “totally agree.” Multimedia Appendix 2 presents the items for each construct and their source.

### Sample Characteristics

Data were collected between November 1 and November 16, 2018 (first company), and between May 15 and May 28, 2019 (second company). The invitation letters and secure hyperlink to the online questionnaire were posted on the intranet (first company) and sent via email (second company). Reminders to participate in the survey were posted in the middle of the data collection periods. Out of 2370 employees, 354 participated in the survey, representing a response rate of 15%. Of these answers, 44 were excluded due to missing data, resulting in a final sample size of 310 responses. In our sample, 178 (57%) participants had used the teleconsultation platform at least once. The remainder of this paper is, therefore, based exclusively on their responses, whereas those employees who have not experienced Dialogue (n=132) by the time of the survey could not contribute toward studying continuance.

## Results

### Profile of the Users and Their Use of the Teleconsultation Platform

The sociodemographic profile of the sampled users is presented in Table 1. Our data set showed that users consulted with Dialogue 3 times on average since registration. Out of the 178 participants, about 43% (n=76) reported using the Dialogue teleconsultation platform three times or more since it became available to them. Such statistics were consistent with the participants’ positive health status, as 68% (n=121) of them rated their health as very good or excellent. Frequency analyses and descriptive statistics of the research variables showed a generally positive perception of Dialogue. For instance, user experience was better than expected for 73% (n=130) of patients, and a majority (n=120, 67%) said that using Dialogue provided more benefits than initially expected. In this regard, saving time and gaining better access to health care resources were the main reasons for using Dialogue, as confirmed by 87% (n=154) and 83% (n=147) of patients, respectively. Unsurprisingly, 79% (n=140) of patients reported that Dialogue met their health needs, and 75% (n=133) said they quickly resolved a health concern using teleconsultation. Furthermore, most patients (n=142, 80%) believed Dialogue helped avoid time away from work.

**Table 1:** Profile of the participants (n=178).

Characteristic		Participants, n (%)
**Gender**		
	Male	47 (26.4)
	Female	129 (72.5)
	Prefer not to answer	2 (1.1)
**Age (years)**		
	18-24	8 (4.5)
	25-34	47 (26.4)
	35-44	66 (37.1)
	45-54	44 (24.7)
	≥55	12 (6.7)
	Prefer not to answer	1 (0.5)
**Education level**		
	Secondary school	6 (3.4)
	College	40 (22.5)
	Certificate	23 (12.9)
	Undergraduate level	74 (41.6)
	Graduate level	33 (18.5)
	Prefer not to answer	2 (1.1)

### Assessment of the Measurement Model

We used structural equation modeling (SEM) to validate the research model and test the research hypotheses, employing the partial least squares (PLS) component-based technique for this purpose. PLS-SEM was chosen because of its robustness and lower requirements regarding the distribution of residuals when compared to covariance-based techniques such as EQS and Amos, in addition to being particularly appropriate when the goal is to explain variance [42]. PLS is also able to handle measurement models that include endogenous formative constructs [43].

Table 2 presents the descriptive statistics and reliability coefficients of the research variables. The first step in the SEM analysis is to simultaneously evaluate the measurement model and the structural model with PLS. One must first note in this regard that a single research construct, namely, the quality of the medical teleconsultation, is modeled as being formative due to the composite and multidimensional nature of its conceptualization [44], whereas the other four constructs are reflective [45]. The measurement model also includes another formative construct, patient characteristics, made up of the three control variables, namely, gender, age, and education level.

**Table 2:** Descriptive statistics and reliability coefficients of the research variables.

Research variable^a^	Items, n	Mean (SD)	Minimum	Maximum	Cronbach alpha^b^	VIF^c,d^
Expectation confirmation	3	3.9 (1.0)	1.0	5.0	.92	N/A^e^
Ease of use	3	4.6 (0.5)	2.3	5.0	.93	1.27
Service quality	3	4.3 (0.8)	1.0	5.0	.92	1.51
Security and confidentiality	4	4.2 (0.7)	2.5	5.0	.95	1.43
Trust	3	4.3 (0.7)	2.1	5.0	.91	N/A
Usefulness	4	4.1 (1.0)	1.0	5.0	.94	N/A
Continuance intention	3	4.4 (0.9)	1.0	5.0	.95	N/A

^a^All variables were measured on 5-point Likert scales (totally disagree to totally agree).

^b^Cronbach alpha coefficient of reliability.

^c^VIF: variance inflation factor.

^d^VIF = 1 / (1 – *R_i_*^2^), where *R_i_*^2^ is the unadjusted *R*^2^ obtained when variable *i* is regressed against all other variables forming a construct.

^e^N/A: not applicable.

The psychometric properties of the measurements were evaluated in the context of the structural model by assessing the unidimensionality and reliability of the research constructs. First, as presented in Table 2, the Cronbach α values of the research variables varied from .91 to .95, all above the .80 threshold and, thus, confirming their internal consistency. Second, the reflective constructs’ item loadings (λ) varied from 0.90 to 0.97, well above the 0.70 threshold and, thus, indicating the unidimensionality of these four constructs. Moreover, the weights (γ) of the three formative variables of the quality construct were all positive and strong enough to be retained in the measurement model [46].

As the usual validity criteria for reflective constructs are inapplicable to a formative construct, one must instead verify that there is no multicollinearity among the formative construct’s indicators. One uses the variance inflation factor (VIF) statistic to do so, a common rule being that a variable’s VIF value be less than 3.3, or in other words, that less than 70% of the variance in the variable be jointly explained by the other variables [47]. As shown in Table 2, the outer VIF values estimated by PLS for the three formative indicators of the quality construct varied from 1.27 to 1.51, well below the 3.3 threshold. In similar fashion, the three formative indicators of the patient characteristics construct had VIF values varying from 1.01 to 1.13, thus indicating the absence of multicollinearity.

In Table 3, one also finds that the composite reliability coefficient of the reflective constructs varied from 0.94 to 0.97, above the 0.70 threshold and, thus, confirming these constructs’ reliability. Their convergent validity was also confirmed, as the average variance extracted ​​(AVE) varied from 0.84 to 0.91, above the 0.50 threshold. The last property to be analyzed in the measurement model, discriminant validity, indicates the extent to which a construct differs from other constructs in the model. In the case of reflective constructs, the shared variance between such a construct and other constructs must be less than the AVE from its indicators, as confirmed in Table 3. In the case of the two formative constructs, quality and patient characteristics, the fact that they shared less than 70% variance with any other related construct in the measurement model, and thus correlated less than perfectly with these constructs, was an indication of such validity [48].

**Table 3:** Reliability, unidimensionality, and discriminant validity of the research and control constructs.

Construct	CR^a,b^	AVE^c,d^	Items’ loading on their respective construct				Interconstruct correlations^e^				
			Item 1	Item 2	Item 3	Item 4	1	2	3	4	5
1. Expectation confirmation	0.95	0.86	0.93	0.93	0.93	N/A^f^	0.93^g^				
2. Quality	—^h^	—	N/A	N/A	N/A	N/A	0.75	N/A			
3. Trust	0.94	0.84	0.92	0.90	0.90	N/A	0.54	0.79	0.92		
4. Usefulness	0.96	0.86	0.92	0.91	0.93	0.95	0.85	0.81	0.63	0.93	
5. Continuance intention	0.97	0.91	0.95	0.94	0.97	N/A	0.70	0.77	0.57	0.82	0.95
6. Patient characteristics	N/A	N/A	N/A	N/A	N/A	N/A	0.11	0.12	0.04	0.11	0.08

^a^CR: composite reliability.

^b^CR = (∑λ_i_)^2^ / ((∑λ_i_)^2^ + ∑(1 – λ_i_^2^)).

^c^AVE: average variance extracted.

^d^AVE = ∑λ_i_^2^ / n.

^e^Subdiagonals: correlation = (shared variance)^1/2^.

^f^N/A: not applicable.

^g^Diagonal: (AVE)^1/2^ = (∑λ_i_^2^/n)^1/2^.

^h^Inappropriate for formative constructs.

### Hypothesis Testing

**Figure 2:**
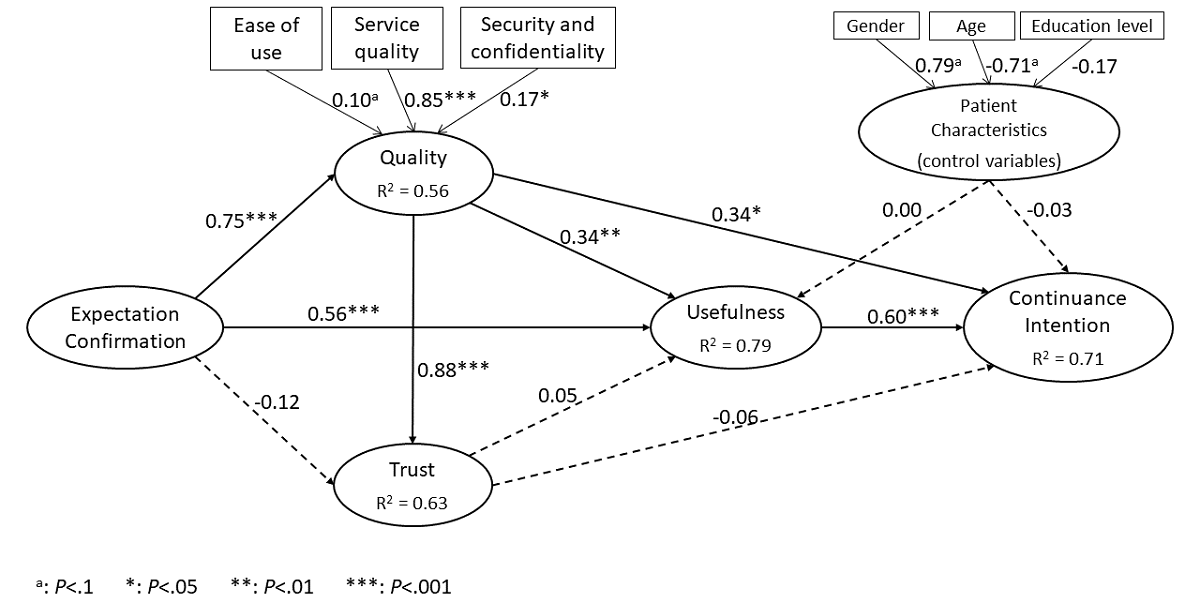
Research findings.

The research model was tested by evaluating the path coefficients (β) that linked the constructs in the research model (using the SmartPLS software, SmartPLS GmbH), as shown in Figure 2. The model’s fit was first assessed by the strength and significance of the path coefficients and the proportion of explained variance in view of PLS’s concern with generalization and focus on prediction [49]. The goodness of fit was also assessed by the standardized root mean squared residual index, whose value was 0.06, well below the 0.08 threshold [43].

#### Hypothesis 1: Confirmed

As seen in Figure 2, a positive and highly significant path coefficient (β=.60, *P*<.001) confirmed that patients who perceive a medical teleconsultation platform to have greater usefulness will have a stronger intent to continue using such a platform. In other words, CI is primarily determined by the benefits patients perceive to have obtained from using teleconsultation. This initial finding confirms Bhattacherjee’s [22] postulate that mHealth IT use is essentially a “means-end” behavior, and this use will continue only if the desired end is achieved.

#### Hypothesis 2: Unconfirmed

A negative and nonsignificant path coefficient (β=–.06, *P*=.27) disconfirmed the hypothesis that patients who place greater trust in a medical teleconsultation platform will have a stronger intent to continue using such a platform. It would thus appear that, in the absence of benefits, patients will not necessarily pursue their use of medical teleconsultation technology even though they trust the technology, its provider, and the medical professionals consulted through it.

#### Hypothesis 3: Unconfirmed

A positive and nonsignificant path coefficient (β=.05, *P*=.27) did not confirm the hypothesis that patients who place greater trust in a medical teleconsultation platform will perceive such a platform to have greater usefulness. Again, it would appear that the patients’ trust in the teleconsultation technology, notwithstanding its presumed criticality in mHealth IT environments, bears no direct consequences upon the personal health benefits obtained from using this technology, nor indirect consequences upon the continuance of such use.

#### Hypothesis 4: Confirmed

The hypothesis that patients who perceive a medical teleconsultation platform to be of greater quality will have a stronger intent to continue using such a platform was confirmed, as the corresponding path coefficient was shown to be positive and significant (β=.34, *P*=.01). Looking at the weights of the three components of quality as conceptualized here, this finding highlights the primary importance of the quality of the support services rendered by the platform’s provider and the teleconsultation exchanges with the platform’s medical professionals (γ=0.85, *P*<.001). Less important but nonetheless determinant are the patients’ perceptions as to the security of the medical teleconsultation platform and the confidentiality of their personal health data (γ=0.17, *P*=.01), as well as their perceptions of the platform as being easy to learn, use, and master (γ=0.10, *P*=.07).

#### Hypothesis 5: Confirmed

A positive and significant path coefficient (β=.34, *P*=.002) confirmed the hypothesis that patients who perceive a medical teleconsultation platform to be of greater quality will perceive such a platform to have greater usefulness. In line with the ECM [18], this finding demonstrates that the personal benefits obtained from using teleconsultation accrue in part from the quality of the remote health services. The security and confidentiality aspects of the medical teleconsultation technology as well as its ease of use also comes into play, however, less importantly.

#### Hypothesis 6: Confirmed

A positive and highly significant path coefficient (β=.88, *P*<.001) confirmed the hypothesis that patients who perceive a medical teleconsultation platform to be of greater quality will place greater trust in such a platform. In line with Akter et al’s [26] findings, it appears that it is the quality of the teleconsultation services that are paramount in gaining the patients’ trust, with the security and confidentiality of the information transmitted through the teleconsultation being of secondary importance in this regard.

#### Hypothesis 7: Confirmed

A positive and highly significant path coefficient (β=.75, *P*<.001) supported the hypothesis that the more the patients’ initial expectations related to using a medical teleconsultation platform are confirmed, the better their perception of the quality of such a platform. The patients’ perceptions of the platform’s quality are deemed to be highly indicative of their affect level [50]. Hence, in line with expectation confirmation theory [18], we found that when confirmation of the patients’ initial expectations increases, so does their appreciation of the quality of mHealth IT and, in turn, their appreciation of their IT use experience.

#### Hypothesis 8: Confirmed

A positive and highly significant path coefficient (β=.56, *P*<.001) supported the hypothesis that the more the patients’ initial expectations related to using a medical teleconsultation platform are confirmed, the better their perception of the usefulness of such a platform. This result is fully in line with a basic proposition of the ECM with regard to the continuance of IT use [22].

#### Hypothesis 9: Unconfirmed

A nonsignificant and negative path coefficient (β=–.12, *P*=.15) did not support the hypothesis that the more the patients’ initial expectations related to using a medical teleconsultation platform are confirmed, the greater the patients’ trust is in such a platform. However, given the preceding results related to hypotheses 7 and 6, it appears that the confirmation of patients’ initial expectations has a significant indirect effect upon their trust in the platform, that is, an effect mediated by their perceptions of the platform’s quality. Thus, notwithstanding the extent to which their expectations are confirmed, patients would not lose trust in a teleconsultation platform if they perceive the platform to be of enough quality.

Returning to Figure 2, the expectation confirmation and quality constructs were shown to jointly explain more than 70% of the variance in usefulness, a *large* effect size [51]. Similarly, quality and usefulness were shown to jointly explain a large proportion of the variance in CI. Moreover, the three control variables were found to provide no added explanation of the patients’ perceptions of the usefulness of the teleconsultation platform nor of their intent to continue using it. Our research model, thus, performed rather well in the nomological integration of the five research constructs. Furthermore, using tests of joint significance of indirect effects [52], we found significant (*P*<.001) and important (percentage of total effects) indirect effects of expectation confirmation on usefulness, that is, through quality (see Table 4). There were also important indirect effects of quality on patients’ CI through usefulness. Finally, we found sizable indirect effects of expectation confirmation on CI, through both quality and usefulness. These last results of the PLS-SEM analysis highlight the “mediating” role played by the patients’ assessment of the medical teleconsultation platform’s usefulness in explaining their decision to pursue or not pursue their use of such a platform.

**Table 4:** Breakdown of the total effects of the research constructs.

Relationships between research constructs	Direct effects	Indirect effects	Total effects
Usefulness → continuance intention	0.587	0.000	0.587
Trust → continuance intention	–0.060	0.028	–0.032
Quality → continuance intention	0.339	0.172	0.511
Expectation confirmation → continuance intention	0.000	0.722	0.722
Trust → usefulness	0.048	0.000	0.048
Quality → usefulness	0.340	0.043	0.383
Quality → trust	0.875	0.000	0.875
Expectation confirmation → usefulness	0.571	0.282	0.853
Expectation confirmation → trust	–0.118	0.657	0.539
Expectation confirmation → quality	0.751	0.000	0.751

## Discussion

### Principal Findings

We examined the influence of usefulness, quality, trust, and expectations confirmation on the CI of a teleconsultation platform in use in Canada. PLS analyses were conducted to test 9 research hypotheses, 6 of which were supported. Based on our findings, the main predictor of teleconsultation CI is perceived usefulness. This is consistent with the extant literature presenting this construct as one of the principal expectations explaining IT continuance [23]. As teleconsultation is exclusively a utilitarian system aiming to provide instrumental value to patients, this finding is also coherent with previous IT use research, which “suggests that utilitarian system-use behaviors are mainly driven by the perceived relevance and usefulness of the system” [53]. Our findings indicate that patients emphasize time savings and quick access to health care professionals as the main benefits. Conversely, the impossibility of remote diagnosis and prolonged waiting times were often presented as the leading causes of low perceived usefulness. In other words, what matters most to patients is that teleconsultation helps them resolve their health issues in an efficient manner.

The inclusion of quality in the research model emerges as an important contribution to the understanding of teleconsultation continuance, considering its significant influence on CI and usefulness. This finding is aligned with extant literature, as several authors observed the influence of quality on users’ behavioral intents [26,34,35]. Along with the quantitative results of this study, the qualitative data obtained through an open-ended question indicated that the leading dimension of perceived quality is service quality rather than confidentiality and security, and ease of use. The high level of interactivity and the object of medical teleconsultations cause patients to be concerned with the quality of their interactions and the medical advice received [54]. Hence, service quality, especially in cases involving patients’ health status, allows for a more sophisticated understanding of the general concept of quality. The operationalization of quality also benefits from the security and confidentiality construct. As argued by Demiris et al [55], “the ethical issues involved with the application of mobile IT to health care are many and complex,” which makes data security and confidentiality a mandatory requirement for teleconsultation systems. Thus, even if it plays a lesser role than the aspect of service, patients perceive a teleconsultation service to be of high quality if their data is protected from theft, loss, and unauthorized use. Finally, the increased ease people experience when using mobile technologies [19] could explain the relatively low importance of perceived ease of use in building a sense of quality. This construct may prove valuable for the study of populations with lower digital literacies than the one covered by this study.

Confirmation of patients’ initial expectations directly influenced teleconsultation usefulness, as expected. Simply put, it is central to determining the usefulness level, especially since it fully accounts for its second drivers (ie, quality) in addition to being its strongest direct predictor. This is captured by the direct and indirect influences of the confirmation of expectations on perceived usefulness reported in Table 4. In addition, expectations confirmation’s role in shaping patients’ intents should not be underestimated in view of its large indirect effect on CI (Table 4). Indeed, it solely explains the variance in quality and usefulness, which were the only two direct significant predictors of our dependent variable. This is critical, as some users did not seem to fully grasp the possibilities and limitations of the teleconsultation platform, as reflected by many of their comments. For instance, some were surprised at being referred for an in-person examination, given the impossibility of remote diagnosis. In view of the novelty of the service under study and the resulting misunderstanding of its constraints, patients’ initial expectations were sometimes unrealistic. Under circumstances where the user experience fell short, usefulness was adversely affected. Therefore, understanding the opportunities and boundaries of online medical consultations is essential for fulfilling expectations, attaining positive levels of usefulness, and sustaining use behavior over time.

Last, in contrast with prior research [26-29], trust was not a significant factor in our model. To explain this unexpected finding, it may be relevant to consider how sensitive the information disclosed by Dialogue users is and how severe the health issues addressed through Dialogue are. Indeed, the data transmitted may not appear sensitive to patients, given the relatively minor nature of the health issues that can be diagnosed through the type of teleconsultation we studied. Nevertheless, trust may be a significant factor, depending on patients’ health issues. For example, it could be relevant in cases of consultation for severe chronic diseases or psychological problems.

### Limitations and Suggestions for Future Research

The results of this study should be interpreted with caution, given some methodological limitations. The size of our sample was relatively small. The context-specific nature of this study requires exercising caution about the generalizability of its findings. Organizations providing similar services to their employees may obtain a different picture than the ones presented here. The same applies to teleconsultations offered to patients by hospitals or clinics. Thus, the advancement of knowledge could benefit from similar studies carried out in other contexts. Use context is partially defined by the technology [56], so other teleconsultation technologies are also worth investigating. Should different teleconsultation contexts be studied, we recommend adapting the operationalization of quality (second-order construct) since dimensions of IS success, like system quality, must be anchored in an empirical context [38].

Given the intrinsic limitations of survey research, there may yet remain biases related to the perceptual nature of the research variables’ measures. In particular, a measurement model based on a self-administered questionnaire answered by a single respondent may pose a risk of common method bias (CMB) and lead to an overestimation of the relationships between variables [57]. We thus used three post hoc techniques to detect the presence of such bias in our data. First, we used the Harman single factor test [57], that is, we made a principal component factor analysis of all scale items, finding that no single component accounted for 50% or more of the aggregate variance in the research variables and, thus, did not detect any moderate to high levels of CMB. Second, we looked at the correlation matrix between the research and control constructs [58], finding all fifteen correlations to be less than 0.90 and, thus, not showing any major sign of CMB. Third, we used Lindell and Whitney’s [59] *marker variable* approach by looking at a variable not assumed to be theoretically linked to any of the research variables. Using the patients’ education level for such a purpose, we found the average correlation of this variable with the seven research variables to be –0.065 (with a minimum of –0.09 and a maximum of 0.04) and, thus, not signaling the presence of CMB in our data.

Another limitation of our study was related to its cross-sectional nature, as causality cannot be inferred. We thus encourage future studies on teleconsultation use continuance to employ longitudinal approaches instead. When undertaken in national health care contexts in which teleconsultation services are not free, future studies should also include socioeconomic variables such as patients’ income, employment, and place of residence [60]. Finally, our results should be interpreted in view of remote health care contexts that could be altered following the resolution of the COVID-19 crisis.

### Contributions of the Study

From a research perspective, our study contributes to the advancement of knowledge by identifying important drivers of patients’ intention to continue using teleconsultation (ie, usefulness, quality, trust, and expectations confirmation). We have also demonstrated that it is appropriate to conceptualize quality as a multidimensional second-order construct, reinforcing previous findings (eg, [34]).

Providers of medical teleconsultation and employers interested in them can benefit from this study. This paper identifies the main predictors of continued use for this type of technology, which is crucial to achieving the expected benefits [22]. Given its key role in developing patients’ CI, usefulness should be monitored frequently through the measurement of actual or perceived benefits arising from teleconsultation use. In this respect, determinants of usefulness should likewise be monitored. Moreover, as usefulness directly and indirectly relies on confirmation of expectations, patients should be provided with clear information about the possibilities, limitations, and potential benefits of online consultations. Patients surveyed in this study expected to have easier access to physicians via teleconsultation, and a few seemed to be frustrated and disappointed that they could not be remotely diagnosed and were referred to in-person services. The deployment of such a system should include establishing an effective communication plan and involving employers, health care providers, and policy makers, depending on the scale of the deployment.

Teleconsultation providers should also try to reduce waiting times and optimize their customers’ health care path since users hope to save time through online medical visits. This is particularly important, as several patients who were instructed to schedule physical examinations felt that the teleconsultation session was a waste of their time. More broadly, our research points to the benefits of focusing on service quality since it is the most important dimension of a teleconsultation’s quality. Some users unable to benefit from remote diagnosis suggested that Dialogue could make the appointments they need for them, so the inconveniences associated with an unsuccessful teleconsultation could give way to a simplification of the appointment scheduling process for other health services. The speed and scope of services and the commitment of providers are appealing avenues for sustaining patients’ continuous use, regardless of their health status.

### Conclusion

This study investigates a technology proving itself to be suitable for public health crisis management [2] as well as being capable of positively addressing challenges of health care systems under normal conditions. We believe that our data set—collected before the COVID-19 pandemic—is more indicative of postcrisis continuance since survey responses collected during the crisis are unlikely to be representative for behavior under normal conditions. We concur that “telemedicine is central to care going forward, not just through this crisis” [14], and is a significant lever for an improved digital delivery of health care in the future. Hence, we hope that our findings will help drive long-term teleconsultation adoption and use, including in the aftermath of the COVID-19 crisis, so that general care improvement and greater preparedness for exceptional situations can be achieved. The teleconsultation service investigated in this study is particularly relevant in this regard as “[i]ncorporating mHealth in health insurance schemes can help solve the cost and payment barriers and encourage not only clinicians' adoption but also patients' use” [61]. As the current public health crisis has revealed that it is possible to address some of the challenges related to remote health care services, we feel hopeful for the materialization of teleconsultation expected benefits in the long run. Future research efforts should continue focusing on virtual care and teleconsultation systems, and investigate how such technologies contribute to collective well-being.

## Appendix

Multimedia Appendix 1 Definitions of constructs.

Multimedia Appendix 2 Operationalization of the research variables.

## Abbreviations

AVE: average variance extracted
CI: continuance intention
CMB: common method bias
ECM: expectation confirmation model
H: hypothesis
IS: information systems
IT: information technology
mHealth: mobile health
PLS: partial least squares
SEM: structural equation modeling
VIF: variance inflation factor
