# Supplementary material for: Investigating Patients’ Intention to Continue Using Teleconsultation to Anticipate Postcrisis Momentum: Survey Study
Source: J Med Internet Res. 2020 Nov 26;22(11):e22081. doi: 10.2196/22081 (PMC7695543; doi:10.2196/22081)
Supplement: Multimedia Appendix 1 [file jmir_v22i11e22081_app1.docx]

## Appendix 1

Definition of the research variables.

| **Definition** | **Source** |
| --- | --- |
| **Continuance Intention**: patients' intention to continue using a medical teleconsultation technology | [22] |
| **Usefulness**: patients' perceptions of the benefits associated with using a medical teleconsultation technology | [26] |
| **Trust**: a patient's willingness to be vulnerable to the actions of another party, say a healthcare professional, when the latter is expected to act in the interests of the former, regardless of the former's ability to monitor or control the latter | [30] |
| **Expectation Confirmation**: patients' perceptions of the congruence between their expectations of a medical teleconsultation technology use and its actual performance | [22] |
| **Service Quality**: patients' perceptions of the level of quality of the service offered through a medical teleconsultation technology | [38] |
| **Ease of Use**: the degree to which patients believe that the use of a medical teleconsultation technology can be done effortlessly | [32] |
| **Security and Confidentiality**: patients' perceptions that their data is protected from unwarranted access and loss, and that it will be used and shared in accordance with their rights | [39] |
