# Supplementary material for: Investigating Patients’ Intention to Continue Using Teleconsultation to Anticipate Postcrisis Momentum: Survey Study
Source: J Med Internet Res. 2020 Nov 26;22(11):e22081. doi: 10.2196/22081 (PMC7695543; doi:10.2196/22081)
Supplement: Multimedia Appendix 2 [file jmir_v22i11e22081_app2.docx]

## Appendix 2

Operationalization of the research variables.

| **Construct** |  | **Measurement items** | **Source** |
| --- | --- | --- | --- |
| **Continuance Intention** (CI) |  |  |  |
|  | CI1 | I plan to continue using the Dialogue platform to ensure my health or that of my partner and my child(ren) | [22] |
|  | CI2 | I do not want to stop using the Dialogue platform |  |
|  | CI3 | I intend to continue using the Dialogue platform in the future |  |
| **Usefulness** (U) |  |  |  |
|  | U1 | Dialogue allowed me to quickly resolve one or more health issues for me or my partner and my child(ren) | [26] |
|  | U2 | Using the Dialogue platform saves me a lot of time (e.g., waiting in the emergency room, the medical clinic, the pharmacy...) |  |
|  | U3 | Using the Dialogue platform meets my health needs |  |
|  | U4 | In general, using the Dialogue platform has proven very useful in my life |  |
| **Trust** (T) |  |  |  |
|  | T1 | I trust all the health professionals at Dialogue | [29] |
|  | T2 | I use the Dialogue app with confidence |  |
|  | T3 | The company Dialogue is worthy of the trust of its users |  |
| **Expectation Confirmation** (EC) |  |  |  |
|  | EC1 | My experience with Dialogue is better than I originally expected | [22] |
|  | EC2 | The use of the Dialogue platform has given me more benefits than originally anticipated |  |
|  | EC3 | In general, my initial expectations related to using the Dialogue platform have been met |  |
| **Quality** (Q) |  |  |  |
| **Service Quality** (SQ) |  |  |  |
|  | SQ1 | In general, the quality of Dialogue as a telemedicine service is high | [38] |
|  | SQ2 | The services offered by the company Dialogue are of good quality |  |
|  | SQ3 | The quality of the exchanges I had with Dialogue's health professionals is high |  |
| **Ease of Use** (EU) |  |  |  |
|  | EU1 | Learning to use the Dialogue platform was easy for me | [32] |
|  | EU2 | Dialogue is an easy platform to master |  |
|  | EU3 | Overall, I find that the Dialogue platform is easy to use |  |
| **Security and Confidentiality** (SC) |  |  |  |
|  | SC1 | My personal data and/or those of my relatives are well protected in the Dialogue platform | [39] |
|  | SC2 | I consider Dialogue to be a secure platform for my personal data |  |
|  | SC3 | Medical consultations performed via the Dialogue platform are confidential |  |
|  | SC4 | Professional secrecy or confidentiality is ensured with each use of Dialogue |  |
